# An ecological-economic model of land-use decisions, agricultural production and biocontrol

**DOI:** 10.1098/rsos.220169

**Published:** 2022-10-05

**Authors:** V. Martinet, L. Roques

**Affiliations:** ^1^ Université Paris-Saclay, INRAE, AgroParisTech, Paris-Saclay Applied Economics, Palaiseau 91120, France; ^2^ Université Paris-Saclay, ENS Paris-Saclay, Centre d'Economie de l'ENS Paris-Saclay, Gif-sur-Yvette 91190, France; ^3^ INRAE, BioSP, Avignon 84914, France

**Keywords:** biocontrol, agroecology, spatial diffusion model, feedback loop, pest, natural enemy

## Abstract

The regulation of agricultural pests by their natural enemies is a key step in the agroecological transition. The level of biocontrol seems, however, to highly depend on the agronomic and ecological context. It is thus important to identify the conditions under which this ecosystem service is efficient as well as the magnitude of its effects. An actual reduction of pesticide use depends on a change in farmers’ decisions, calling for the consideration of economic dimensions. We develop a dynamic agroecological-economic model representing land-use and agricultural intensity decisions as well as the dynamics of a crop pest and a natural enemy. Biocontrol is assessed considering both private benefits (increase in farmers’ profit) and public benefits (reduction of pesticide use) with respect to a situation without a natural enemy. We provide a theoretical assessment of the magnitude of biocontrol over a wide range of agronomic contexts (spatially explicit maps of agricultural production potential, with heterogeneous distribution and control of spatial fragmentation) and ecological contexts, described through various parameter values of a reaction–diffusion model. The contexts in which biocontrol plays a significant role are identified, and the role of key parameters is discussed. Our open-access model offers a tool to investigate alternative specifications.

## 1. Introduction

The intensification of agricultural practices and the simplification of agricultural landscapes have resulted in biodiversity loss in agroecosystems [1,2] and the reduction of the ecosystem service of natural pest control [3]. In the meanwhile, pest populations have been managed through the use of pesticides, resulting in increased pollution and a social demand to reduce their use. Agroecological approaches, such as a reduction of intensity in croplands and increased diversity of agricultural landscapes with the inclusion of non-crop habitats (NCHs) are presented as a way to foster conservation biocontrol to protect crops [3]. The response of natural enemy populations to landscape diversity is not consistent, however, and does not necessarily result in pest control or reduced damage to crops [4–6], limiting the adoption of conservation biocontrol as a pest control strategy.

Is conservation biocontrol an efficient strategy to manage pests? How to assess its potential to protect crops and achieve an actual reduction of pesticide use in different contexts? A larger population of natural enemy may not result in the effective biocontrol of a pest, as the actual ecosystem service will depend on the population of predators, but also on their ability to colonize cropland and to regulate the pest population [3,4]. Private and social benefits of biocontrol will also depend on the changes in crop-protection behaviour and the associated reduction in pesticide use, meaning that the issue is not only agroecological but also economic, with a need to account for farmers’ decisions. Management strategies based on conservation biocontrol are more likely to be adopted if they result in effective crop protection and are profitable. Whereas the literature has focused on the identification of the ecological drivers of biocontrol [7], few studies consider the actual economic benefit of biocontrol [6].

In this paper, we aim to estimate the magnitude of the effect of biocontrol as well as determining some elements of the contexts in which biocontrol could contribute significantly to the management of crop pests. For this purpose, we develop a dynamic agroecological-economic model representing land-use and agricultural intensity decisions as well as the ecological dynamics of a crop pest and a natural enemy. Annual farmers’ decisions are driven by profit maximization, accounting for local pest pressure, resulting in a dynamic landscape. Comparing a situation with and without a natural enemy, the biocontrol ecosystem service is assessed at the landscape level through (time-average) profit variation to account for private benefits, and pesticide use reduction to account for social benefits.

One aspect common to most models of interacting species is that the results strongly depend on the parameters, whether these parameters are related to the landscape [8,9] or to biotic interactions [10]. As a result, the question of the efficiency of biocontrol requires testing a large number of sets of admissible parameters, and the answer will not be unique but rather give ranges of variation and trends. We thus consider a range of agronomic contexts, described as spatially explicit maps of agricultural production potential, with heterogeneous distribution and spatial configuration, as well as a range of ecological contexts, described through various parameter values of a reaction–diffusion model.

Several research articles studied the drivers of biocontrol, either in terms of landscape composition and the spatial distribution of NCHs [3], or by considering crop-protection practices (e.g. interactions between pest management in organic and conventional farming systems within a landscape-scale model of parasitoid–host interactions [11]). These landscape drivers interact with ecological drivers such as dispersal abilities or the degree of specialization of the natural enemy [3], creating environmental filters that can influence natural enemy communities and the level of biocontrol through the selection of traits [7]. These interactions can occur at the local (field and field-edges) and global (landscape) scales [4]. Few studies, however, address the trade-off with production or the economic valuation of the biocontrol ecosystem service [5], suggesting that research should focus on the link between landscape composition and reduced damage, increased yield and improved profit. The economic value of biocontrol can be assessed at the individual level, estimating the private costs and benefits for a farmer, at a landscape level, accounting for the costs and benefits of a group of farmers whose outcome depends on the interactions among individual decisions influencing (mobile) pest and natural enemy, or at the global level, accounting for the social costs and benefits of crop protection [12].

Our modelling approach makes it possible to provide an economic value of the biocontrol service at all these scales. Modelling farmers’ decisions and their economic and ecological drivers, we can analyse the complex interactions within agroecological-economic systems, accounting for ecological feedback on farmers' land use and agricultural intensity decisions. Our model is relatively simple, still and focuses on stylized patterns, mainly representing ecological traits [7], allowing us to illuminate core dynamic interactions and identify key drivers [13,14]. It offers results that complete approaches based on data and statistical analysis to assess pest and natural enemy abundance, predating rates and crop damage as a function of landscape composition [5]. Other spatially explicit mechanistic simulation models have been designed to study biocontrol in agricultural landscapes, but, following the landscape ecology approach [15], the landscape was generally a given of the agroecological models, thereby neglecting endogenous landscape dynamics based on individual farmers decisions accounting for economic aspects and ecological feedback loops [11]. As emphasized by [16], an important challenge is to develop feedback models that incorporate spatio-temporal interactions between landscape, ecological processes and stakeholder decisions. Despite their importance, such models, which have proved useful for studying conservation policies [17], the evolution of resistance to pesticides [18] and ecosystem services provision [19], are still rare.

## 2. Material and methods

### 2.1. Model overview

We propose a global spatio-temporal model, including the simulation of the soil quality map, the integrated simulation of pest and natural enemy dynamics and of agricultural land-use decisions. It accounts for feedback loops between ecological and economic compartments. The stochastic soil quality map model allows the generation of a large number of scenarios, with precise control of average quality and spatial auto-correlation (or, on the contrary, of fragmentation). The ecological part of the model describes the dynamics of a population of crop pest and of their natural enemy, which depends on land uses and pesticide application. The economic part of the model describes farmers’ land use and production decisions, i.e. (i) how they allocate land to crop production, or leave it as a NCH, e.g. grassland, meadow or set-aside; (ii) how they optimize the use of fertilizers on crops; and (iii) how they define the degree of pest control through pesticide use. These decisions depend on agronomic conditions (encompassed in a local soil quality index), economic conditions (including production price for agricultural outputs, as well as inputs and production costs) and ecological conditions resulting from the presence of pests and their natural enemy. The model computes various indicators to assess the local (at the field level) and regional (at the landscape level) agronomic, economic and environmental performances, at each time. In particular, it makes it possible to provide a theoretical assessment of the magnitude of biocontrol.

### 2.2. Agronomic context: a stochastic soil quality map model

The model represents an agricultural landscape composed of different plots that each can be used either as a cropland or as a NCH. This landscape is represented on a lattice Ω of dimension n×n, which makes it possible to apply the model to raster data. Each cell $x=(i,j)\in {\left[1:n\right]}^{2}$ represents 1 ha, and is characterized by a single quality parameter Q(x), which represents the potential yield of the plot if used as a cropland, i.e. the maximal yield that could be reached when there is no limiting factor (e.g. with maximal fertilization) and in the absence of pest damage. This quality parameter takes value in a range that may depend on the studied region and related crop type. We consider the values $Q_{\min}=0$ and $Q_{\max}=12$ (tons ha^−1^), which correspond to realistic minimal and maximal yields for cereal crops in temperate regions [20]. Soil quality can be more or less correlated across space. A given agronomic context can thus be described by a distribution of soil quality within the range $\left[Q_{\min};Q_{\max}\right]$ along with a parameter describing the spatial auto-correlation.

To analyse contrasted cases, we generate random virtual maps based on random soil quality distributions (truncated normal distributions characterized by different mean and variance values) and spatial auto-correlation levels. Each generated map corresponds to a virtual agricultural region of more or less good quality, more or less heterogeneous, and with more of less spatial auto-correlation. This allows us to study a diversity of agronomic contexts, and the effect of landscape configuration on the performance of agroecological systems. More precisely, to build a quality map Q(x) defined on the lattice Ω, we proceed in two steps.

*Step 1: Generation of the values in Q.* We begin by drawing $n^{2}$ values in a truncated normal distribution, within $\left[Q_{\min};Q_{\max}\right]$. This distribution has two parameters. The first parameter is the mode of the distribution (the mean value of the Gaussian before truncation) $\bar{q}$. Higher values correspond here to regions with a higher agricultural potential. The second parameter is the standard deviation $s_{q}$. Higher values of this parameter mean more variable soil qualities. This generates a vector $Q^{*}$ of $n^{2}$ values in $\left[Q_{\min};Q_{\max}\right]$ with no *a priori* on the spatial distribution of these values.

*Step 2: Spatial arrangement of the quality map Q(x).* In a second step, we use a stochastic model to build spatial arrangements of these quality distributions. Our approach is an extension of the neutral landscape generator MULTILAND [21] intended for theoretical studies on the effect of landscape structure in applied sciences (see also [22] for a review on landscape modelling approaches). We consider that the lattice Ω is equipped with a four-neighbourhood system with periodic conditions. In other terms, the domain is considered wrapped on a torus. We denote by $V_{x}$ the set containing the four neighbours (i+1,j), (i−1,j), (i,j+1) and (i,j−1) of x=(i,j). Given any spatial arrangement Q of the $n^{2}$ values in $Q^{*}$, we define the statistic

2.1 $$S(Q)=\frac{C}{4}\sum_{x\in \Omega ,y\in V_{x}}|Q(x)-Q(y)|,$$

for a given positive constant C (C=10 in our simulations). The statistic S(Q) is directly linked to soil quality fragmentation. Large values of S(Q) indicate that neighbours tend to be different (higher fragmentation), while low values indicate that neighbours tend to resemble each other (low fragmentation). The landscape model is based on the Gibbs measure P, defined over all the possible arrangements of the values in Q*:

2.2 $$P(X=Q)=\frac{1}{Z}\;e^{\;f\;S(Q)}\text{ with the partition function }Z=\sum_{\tilde{Q}\text{ with values in }Q^{*}}\;e^{f\;S(\tilde{Q})}.$$

**Figure 1.**
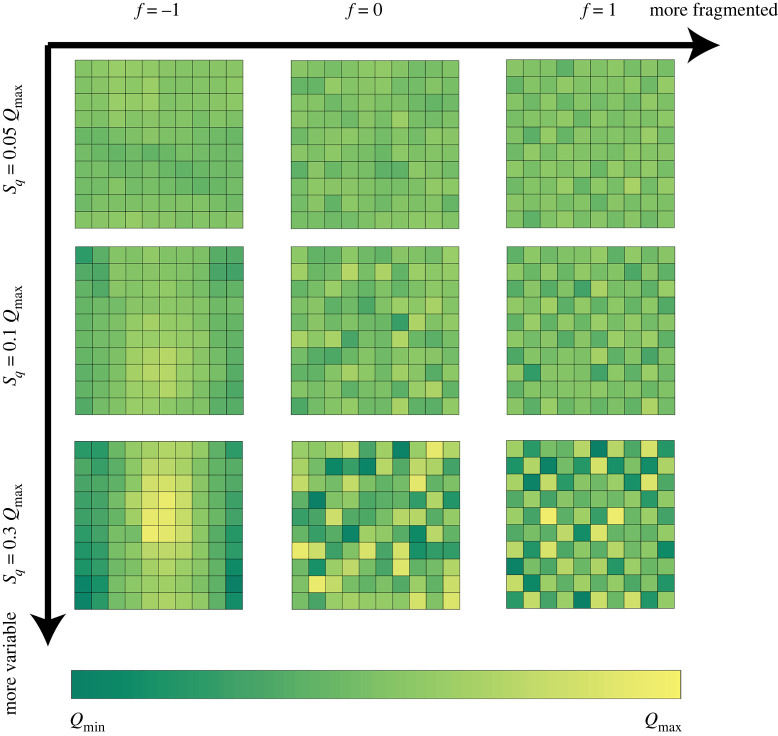
Soil quality maps simulated with varying values of the fragmentation and variability parameters.

When f increases, the proposed landscape model favours higher values of S(Q), i.e. higher fragmentation: given two arrangements $Q_{1}$ and $Q_{2}$ of the values in $Q^{*}$, with $S(Q_{1})>S(Q_{2})$, the relative probability $P(X=Q_{1})/P(X=Q_{2})=\exp[\;f(S(Q_{1})-S(Q_{2}))]$ becomes larger as the parameter f is increased. Hence, f∈R directly controls the topology of the landscape patterns. We refer to it as the *fragmentation parameter*. Typically, negative values of f lead to aggregated maps and positive values lead to fragmented quality maps. We give several examples of quality maps generated with this model in figure 1.

### 2.3. Ecological compartment: a lattice dynamical system with spatial diffusion

We use a lattice dynamical system to describe the intra-annual spatio-temporal dynamics of a pest together with their natural enemy over the landscape Ω, i.e. the finite grid of dimension n×n. Lattice dynamical systems are the discrete-space counterparts of reaction–diffusion models, which are themselves among the most popular models to describe the spatio-temporal dynamics of interacting species in heterogeneous environments (e.g. [23–27] for reference textbooks). Here, we do not need to describe the spatial dynamics of the species *within* each cell of the map, which would be unnecessarily time-consuming for our purpose, hence the use of a discrete-space model adapted to the lattice Ω.

We denote by τ the time since the beginning of the simulation. The (continuous) time τ is expressed in years. We also define a discrete year index t∈N by t=⌊τ⌋ (⌊⋅⌋ being the floor function).

We denote by $P_{\tau }(x)$ the pest density at time τ and in the cell x and by $N_{\tau }(x)$ the density of natural enemy. The lattice dynamical system governing the species dynamics and their interactions is

2.3 $$\begin{array}{cc} & P_{\tau }^{\prime }(x)=D_{P}D[P_{\tau }(x)]+g_{P}(\tau ,x,P_{\tau }(x))-\alpha P_{\tau }(x)N_{\tau }(x)-\varphi_{\tau }(x)P_{\tau }(x),\\ & N_{\tau }^{\prime }(x)=\underset{\mathrm{dispersal}}{\underbrace{D_{N}D[N_{\tau }(x)]}}+\underset{\mathrm{growth}}{\underbrace{g_{N}(x,N_{\tau }(x))}}+\underset{\mathrm{interactions}}{\underbrace{\alpha P_{\tau }(x)N_{\tau }(x)}}-\underset{\mathrm{pesticide}}{\underbrace{\varphi_{\tau }(x)N_{\tau }(x)}}. \end{array}\}$$

Here, the sign (′) denotes the derivative with respect to time. We now detail the components of these equations.

*Dispersal.* We describe the movements of the individuals with a discrete Laplace operator (discrete-space counterpart of the standard Laplace diffusion), corresponding to uncorrelated random walk movements of the individuals over the lattice Ω. For x=(i,j) and any function U(x)=U(i,j) defined over the lattice Ω,

2.4 $$D[U(i,j)]=\frac{U(i+1,j)+U(i-1,j)+U(i,j+1)+U(i,j-1)-4U(i,j)}{\delta_{x}^{2}},$$

with $\delta_{x}=L/n$, L the width of the study site fixed here at L=1 (without loss of generality, up to scaling in the space variable). The diffusion coefficients $D_{P}$ and $D_{N}$, respectively, measure the mobility of the pest and of its natural enemy. We assume periodic conditions at the boundary of the lattice, meaning that the study site is embedded in a larger periodic landscape with the same species dynamics over each period cell.

*Growth.* The functions $g_{P}$ and $g_{N}$ describe the growth (or decline) of the pest and natural enemy populations, in the absence of interactions between these two species and pesticide treatments. Regarding the pest dynamics, we assume that the growth is zero during the first half of the year, corresponding to the months following the harvest, which occurs at each discrete time t∈N. The modelling time starts with the beginning of the cultural season (corresponding to the harvest of the previous crop cycle) and the time period is that of the crop cycle. For simplicity, we assume it is a full calendar year, but the model could use a different time period to accommodate the case of multiple harvesting per year. It would require adjusting the parameter values.

During the second half of the year, we assume a standard logistic growth, with parameters which depend on the land use and on the quality parameter at the current position x,

2.5 $$g_{P}(\tau ,x,P_{\tau }(x))=\left\{ \begin{array}{cc} \;0 & \;\text{if }\tau \in \left[t,t+\left(\frac{1}{2}\right)\right),\\ R_{P,\tau }(x)P_{\tau }(x)\left(1-\frac{P_{\tau }(x)}{Q(x)}\right) & \text{if }\tau \in \left[t+(\frac{1}{2}),t+1\right). \end{array}\right.$$

The intrinsic growth rate $R_{P,\tau }(x)$ takes a positive value ($R_{P,\tau }(x)=r_{P}>0$) over cultivated cells and is nil ($R_{P,\tau }(x)=0$) over NCH. It, therefore, depends on the current land use in the cell x at time τ. On cultivated cells, and during the second half of the year, we make the assumption that the maximum number of pests that can be reached in a cell x before saturation is proportional to the potential yield Q(x). This makes sense, as the potential yield Q(x) was precisely defined as the maximal yield that could be reached in the absence of pests (and without other limiting factors). Up to a scaling factor, meaning here that we change the unit in which the pest population is measured, the carrying capacity for the pest is simply Q(x).

Regarding the natural enemy dynamics, we assume that the natural enemy does not reproduce on crops. Their dynamics are thus driven by death only, with a rate 1/γ, γ being the natural enemy life expectancy. On NCH, we assume a logistic growth with intrinsic growth rate $r_{N}>0$. Up to a renormalization (the population of natural enemy is expressed in proportion of the carrying capacity), the carrying capacity is fixed at 1. Overall, we thus assume the following form for the function $g_{N}$:

2.6 $$g_{N}(x,N_{\tau }(x))=\left\{ \begin{array}{cc} \;\frac{-N_{\tau }(x)}{\gamma } & \text{on crops},\\ r_{N}N_{\tau }(x)(1-N_{\tau }(x)) & \text{on NCH}. \end{array}\right.$$

*Interactions.* We assume standard Lotka–Volterra interactions between the pest population and its natural enemy, which means that the pest death rate increases linearly with the density of natural enemy, and conversely the growth rate of the natural enemy increases linearly with the pest population density. For simplicity, we assume a ‘one-to-one’ relationship, i.e. the population of natural enemy is increased by one unit when one unit of pest population is consumed, with the same interaction factor α summarizing predation efficiency.

*Pesticide effects.* We assume that pesticide treatments increase the death rate of both species in the same way. Again, this is a simplifying assumption: it is known that the natural enemies are often sensitive to pesticides [28], but the impact on the death rate may not be identical for the two species. The term $\varphi_{\tau }(x)$ takes the value 0 in the absence of treatment (NCH and non-treated crops). On treated crops, we assume a baseline mortality rate $\varphi_{\tau }(x)=\rho >0$ on slightly treated crops, which is doubled in highly treated crops: $\varphi_{\tau }(x)=2\rho$.

A fundamental issue is to determine as realistic as possible values for the parameters $D_{P}$ , $D_{N}$, $r_{P}$ , $r_{N}$, γ, ρ and α. Of course, they depend on the considered species and pesticide. In electronic supplementary material, S1, we determine reasonable orders of magnitude, with simplified models where each mechanism is considered independently. The corresponding values are summarized in table 1.

**Table 1.** Summary of the main notations and parameter values.

notation	description	values	unit	source
$\bar{q}$	mode of the soil quality distrib	$Q_{\max}\times \{0.3,0.5,0.7\}$	${\mathrm{ton}\;\mathrm{ha}}^{-1}$	
$s_{q}$	s.d. of the soil quality distrib	$Q_{\max}\times 0.05,0.1,0.3$	${\mathrm{ton}\;\mathrm{ha}}^{-1}$	
f	fragmentation parameter	−1,0,1	dimensionless	
$D_{P}$	pest diffusion coeff.	$(1/n^{2})\times 0.1,1$	${\mathrm{km}}^{2}y^{-1}$	electronic supplementary material, S1
$D_{N}$	natural enemy diffusion coeff.	$(1/n^{2})\times 0.1,1$	${\mathrm{km}}^{2}y^{-1}$	electronic supplementary material, S1
$r_{P}$	pest growth rate	$\ln(4),\ln(10^{2}),\ln(10^{4})$	$y^{-1}$	electronic supplementary material, S1
$r_{N}$	natural enemy growth rate	ln(2)	$y^{-1}$	electronic supplementary material, S1
γ	life expectancy of N in a crop	1/2	y	electronic supplementary material, S1 and [29]
α	P−N interaction term	0,1/3,5/6,4/3	$N^{-1}y^{-1}$ or $P^{-1}y^{-1}$	electronic supplementary material, S1
ρ	baseline pesticide-induced mortality rate	$\ln(4),\ln(10^{2}),\ln(10^{4})$	$y^{-1}$	electronic supplementary material, S1
$C_{C\to \mathrm{NCH}}$	NCH set-up cost	219.4	${\mathrm{euros}\;\mathrm{ha}}^{-1}y^{-1}$	[30]
$C_{\mathrm{NCH}\to C}$	cropland set-up cost	27.4	${\mathrm{euros}\;\mathrm{ha}}^{-1}y^{-1}$	[30]
$s_{\mathrm{NCH}}$	return on NCH	300	${\mathrm{euros}\;\mathrm{ha}}^{-1}y^{-1}$	value inspired from MAEC subsidies
$c_{1}$	share of potential yield depending on fertilization	0.38	none	[31]
$c_{2}$	marginal effect of fertilization on yield	0.015	${\mathrm{ha}\;\mathrm{kg}}^{-1}$	[31]
p	agricultural output price	150	${\mathrm{euros}\;\mathrm{ton}}^{-1}$	value inspired from AGRESTE data for main field crops
$\lambda_{\phi }$	fertilizer cost	1.62	${\mathrm{euros}\;\mathrm{kg}}^{-1}$	computed from AGRESTE data for a mix (N,K,P) = (3,1,1)
$\lambda_{\varphi }$	pesticide cost	33	${\mathrm{euros}\;\mathrm{IFT}}^{-1}$	[32]
ν	cropland fix costs	110	${\mathrm{euros}\;\mathrm{ha}}^{-1}y^{-1}$	computed from AGRESTE data (seeds, insurances, fuel).

### 2.4. Economic decision model

The economic part of the model is a dynamic multi-agent model (cellular automaton) in discrete time. It determines, for each time period [t,t+1), local land use and agricultural practices according to an optimization pattern.

Each cell of the lattice can be allocated either to a representative crop or to NCH. These two uses generate different returns, reduced by set-up costs when applicable. We consider an asymmetric land-use change costs C, with a higher cost to set up a NCH with ecological benefits (sowing and management costs) than to overturn it (tillage and herbicide use), i.e. $C_{C\to \mathrm{NCH}}>C_{\mathrm{NCH}\to C}>0$. In our formalism, NCHs include grassland, meadows and set-aside. In practice, land-use changes between crop and NCH are less frequent than changes between crops, but are important from an ecological point of view. NCH are frequently converted to cropland. Conversely, croplands can be converted to temporary grassland, meadows or set-aside, in particular when market prices do not provide enough revenue for cropland, or when public policies provide incentives for such land cover change (see [33] for global land cover change data, and in particular fig. 4.6 therein, which illustrates the asymmetric pattern).

All economic values are expressed per hectare (ha) to avoid scale effects. We thus implicitly assume a constant return to scale on all fixed production factors (labour, capital, etc.) other than land. There are decreasing returns to scale on land, due to the heterogeneous soil quality [20].

*Farmers' rationality, anticipations and decisions.* We assume that the decisions in each cell mimics a rational farmer maximizing profit. To represent the fact that crop protection through the use of pesticide is more a discrete decision regarding the frequency of treatments than a decision on a treatment quantity over a continuous range, we consider three possible levels of treatment: no treatment at all, an average-treatment level and a high-treatment level. The variable $\varphi_{t}$ can thus take only three values (0, ρ or 2ρ). In terms of calibration, we inspire our parameter values from the treatment frequency index (TFI) for main field crops in France [34], considering a moderate level of treatment at 3 unit-dose ha^−1^ yr^−1^, and a high level at 6 unit-dose ha^−1^ yr^−1^. As a consequence of this modelling choice, at the beginning of the period (representing a cultivation year), the farmer has four distinct choice options corresponding to land-use and crop protection combinations, i.e. NCH, untreated cropland, moderately treated cropland and highly treated cropland. For each of these options, optimal fertilizer application can be determined and an anticipated profit computed (see the details below). Then, the farmer selects the optimal use of the plot, which also defines the optimal pesticide use, by choosing the profit-maximizing option. The model thus determines the land use and pesticide application for all plots (or equivalently, cells), offering a starting point for the modelling of the ecological annual dynamics.

Such a local decision pattern relies on anticipation of local pest population and associated damage under the four potential land-use and pest-control options. This anticipated local pest population is denoted by ${\tilde{P}}_{t+1}(x)$. This is the population accounted for in profit maximization and land-use decisions. The local population will depend on the ecological dynamics of pest and natural enemy populations in the landscape, and thus not only on local decisions. Anticipating this population in practice would be a very difficult task for two reasons. First, it would require to know in advance the exact composition and structure of the landscape, which would result from the separate choices of all farmers. Second, it would require to have perfect knowledge of ecological populations’ size across the whole landscape and of ecological spatial dynamics. To avoid heroic assumptions on farmers’ ability to foresee these dynamics, we assume that the farmer accounts only for local pest growth given initial pest population in the field, without accounting for future predation and diffusion. This means that biocontrol has an *ex post* effect, through past predation and a lower pest population, but is not accounted for *ex ante*. This assumption also implies that the anticipation does not depend on other farmers’ decisions, allowing us to abstract from game-strategic considerations arising from simultaneous decisions among interacting agents [35]. Overall, this means that ${\tilde{P}}_{t+1}(x)$ is the solution at time t+1 of the simplified model

$${\tilde{P}}_{\tau }^{\prime }(x)=g_{P}(\tau ,x,{\tilde{P}}_{\tau }(x))\text{ with initial condition }{\tilde{P}}_{t}(x)=P_{t}(x).$$

*Return on cropland.* The return on cropland depends on local yield $Y_{t}(x)$, crop price p, input costs for fertilizers ($\lambda_{\phi }$) and pesticides ($\lambda_{\varphi }$), fixed costs ν and potential conversion costs $C_{\mathrm{NCH}\to C}$ if the plot was used as a NCH at the previous time period. We assume that the representative crop price is constant across time, but global market effects could be included, for example, by modelling price fluctuation with a dynamic system of auto-correlated prices with exogenous random shocks, and assuming that farmers have rational anticipations, as in [36].

Cropland profit thus reads

2.7 $$\pi_{t}^{C}(x)=\;p\;Y_{t}(x)-\lambda_{\phi }\phi_{t}(x)-\lambda_{\varphi }\varphi_{t}(x)-\nu -1_{\mathrm{NCH}\to C}C_{\mathrm{NCH}\to C},$$

where $1_{\mathrm{NCH}\to C}$ is equal to 1 if the plot was a NCH at time t−1, and 0 otherwise.

*Crop yield and pest damage.* Croplands have a yield $Y_{t}(x)$ which is heterogeneous across space, depending on the local soil quality Q(x), but also on the use of fertilizers $\phi_{t}(x)$ and on damage L from pests (yield loss) depending on local pest population $P_{t+1}(x)$ at the harvest time t+1 (end of the economic year [t,t+1)). As the pest population is mobile, this damage depends both on local decisions (pesticide use) but also on land uses and pesticide use across the whole landscape, as well as on the presence of a natural enemy.

To model agricultural production, we consider a yield function of the ‘Mitscherlich–Baule’ type [37–39] augmented of pest damage,

2.8 $$Y_{t}(x)=Q(x)(1-c_{1}\;e^{-c_{2}\phi_{t}(x)})(1-L(x,P_{t+1}(x))).$$

The parameter $c_{1}$ represents the share of potential yield linked to fertilization, and the parameter $c_{2}$ determines the marginal effect of fertilization on yield. These two parameters are exogenous and depend on the crop type. Farmers determine the optimal level of fertilizer to be applied in any treatment option by maximizing profit with respect to the decision variable ϕ given anticipated pest populations ${\tilde{P}}_{t+1}(x)$. Given the equations for yield and profit, standard conditions lead to the optimal value

2.9 $$\phi_{t}(x)=\frac{-1}{c_{2}}\ln\left(\frac{\lambda_{\phi }}{\;pc_{1}c_{2}Q(x)(1-L(x,{\tilde{P}}_{t+1}(x)))}\right).$$

The term $L(x,P_{t+1}(x))$ corresponds to the production loss due to pest damage, which we assume to depend on the local pest population at the end of the period, i.e. at the harvest time. Other assumptions could have been considered as well, depending on the type of pest, for instance, the production loss may depend on the cumulated number of pests during the cultivation period. Several damage functions have been proposed in the literature. For example, [40] uses the functional form aP/(1+bP), in which the parameters a and b are related to the virulence of the pest. The limit of this type of damage function is that it does not take the link between the production level and the carrying capacity of the pest in the plot into account, and how this link affects the damage level. Assuming that the carrying capacity of the pest is proportional to the production level, and that this carrying capacity is reached when damage is maximal (full crop destruction), we assume here that damage is proportional to the density of the pest, i.e.

$$L(x,P_{t+1}(x))=\frac{P_{t+1}(x)}{Q(x)},$$

where we recall that the carrying capacity of the pest is proportional to the production level, and that this carrying capacity is reached when damage is maximal (full crop destruction), leading to $L(x,P_{t+1}(x))=1$ when $P_{t+1}(x)=Q(x)$. With this representation of damage, pest virulence is directly linked to the pest growth rate over the cultural period. Note that the level of pesticide use does not appear explicitly in the expression, but affects yield indirectly through the local pest density reduction, and thus reduces losses.

*Return on NCH.* NCHs generate a return $s_{\mathrm{NCH}}$ (e.g. an agroenvironmental subsidy), so that the profit for this land use is

2.10 $$\pi_{t}^{\mathrm{NCH}}(x)=s_{\mathrm{NCH}}-1_{C\to \mathrm{NCH}}C_{C\to \mathrm{NCH}},$$

where $1_{C\to \mathrm{NCH}}$ is equal to 1 if the plot was cropland at time t−1, and 0 otherwise. This return does not depend on soil quality, nor on the pest population.

### 2.5. Parameter values and simulation plan

Our simulations aim at assessing the performance of different agricultural landscapes, and the way it is influenced by biocontrol. For this purpose, we consider different contexts. The agronomic context is described by three parameters: the mean soil quality, its variation and the fragmentation index. The ecological context is described by six parameters: the pest diffusion coefficient, the natural enemy diffusion coefficient, the pest growth rate, the natural enemy growth rate, the natural enemy life expectancy in a crop and the predation rate. The economic context only varies with respect to the efficiency of pesticide, i.e. the induced mortality rate. We explain how the model can be used with varying economic contexts in the discussion.

We detail in table 1 the full list of parameters of the whole agronomic-ecological-economic model.

We considered a relatively large number of reasonable contexts: 27 agronomic contexts × 48 ecological contexts × 3 levels of pesticide-induced mortality rate. This led to 3888 contexts.

Though the ecological and economic compartments are deterministic, the overall model is stochastic due to the soil quality map part. For each set of parameter values $\left(\bar{q},s_{q},f\right)$ we generated eight quality maps Q. For each map, we simulate the economically driven landscape in the absence of pest, and what happens when the pest is introduced. The idea is to assess the resilience of the landscape to the pest invasion, given the population of natural enemy in the landscape. For each simulation, we initialized the ecological model by assuming that the density of natural enemy was 1 (i.e. equal to the carrying capacity) in the NCH. In an attempt to reduce the effect of this initial choice, we let the system evolve without pest during 3 years. Then, the pest was introduced at the end of the third year, at a density 0.2Q(x) (20% of the carrying capacity) and the model was simulated over T=10 additional years.

Altogether, as we generated eight repetitions for each of the 27 agronomic contexts and simulated the ecological-economic model for each set of parameters, we ran 31 104 simulations, a quarter of which corresponds to the absence of natural enemy (when the predation rate is zero).

### 2.6. Performance indicators

To assess the performance of the agricultural landscape in the different scenarios, we define the mean profit (denoted by π) and the mean treatment frequency index (denoted by TFI) for pesticide use, both expressed per ha and per year. These two quantities represent two important outcomes for society, the value of agricultural production and the level of chemical pollution by pesticides. They are defined as mean values over space and time. Spatial average makes it possible to account for the dual effect of NCH, which reduce the cultivated area but contribute to crop protection by supporting biocontrol. Temporal average smooths temporal fluctuations. The indicators are computed from the introduction of the pest in the landscape to the simulation horizon, corresponding to T years (T=10 in our simulations).

2.11 $$\pi =\frac{1}{T}\sum_{t=1}^{T}\frac{1}{n^{2}}\sum_{x\in \Omega }\pi_{t}(x)\;\text{and}\;\mathrm{TFI}=\frac{1}{T}\sum_{t=1}^{T}\frac{1}{n^{2}}\sum_{x\in \Omega }{\text{TFI}}_{t}(x),$$

where $\pi_{t}(x)$ and $TFI_{t}(x)$ depend on the land use. In particular $TFI_{t}(x)=0$ in NCH and non-treated crops, $TFI_{t}(x)=3$ units in moderately treated crops, and $TFI_{t}(x)=6$ units in highly treated crops.

For scenarios with no predation (α=0, or equivalently N=0), which corresponds to an absence of biocontrol, we denote these quantities by $\pi^{0}$ and $TFI^{0}$. They are used as a benchmark to assess the effect of biocontrol.

Given these mean profit and TFI, for each simulation scenario, we are able to compute two indicators related to biocontrol. Our first indicator measures the profit gain in the presence of biocontrol compared with the absence of biocontrol

2.12 $$\Delta \pi =\pi -\pi^{0}.$$

Our second indicator measures the reduction in TFI in the presence of biocontrol compared with the absence of biocontrol

2.13 $$\Delta \mathrm{TFI}={\mathrm{TFI}}^{0}-\mathrm{TFI}.$$

These two indicators represent the social benefits of the biological ecosystem services, composed of an economic gain on agricultural production and a reduced pollution due to pesticide savings. Note that they are defined such that positive values correspond to a benefit.

## 3. Results

**Figure 2.**
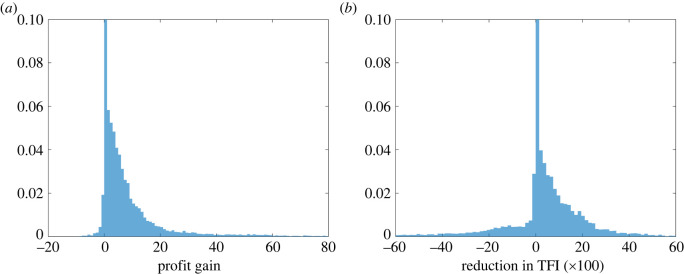
Marginal distributions of the profit gain and the reduction in TFI, compared with an absence of biocontrol. (*a*) Δπ and (*b*) 100×ΔTFI.

*Global effects of biocontrol.* We depict in figure 2 the distributions of the profit gain Δπ and of the reduction in TFI, ΔTFI, compared with the absence of biocontrol. The mean profit gain is $5.7eurosha^{-1}$ (corresponding to 1.3% of mean profit) and the 95% interquartile range (−0.1,43.6). The mean reduction in TFI is $1.2\times 10^{-2}$ units (corresponding to 0.7% of mean TFI) and the 95% interquartile range for reduction in TFI (−0.4,0.3). The average effect of biocontrol is thus relatively low when parameters are chosen at random within the ranges we explored.

These distributions are computed over the whole simulated dataset. Of course, they can change if we consider a subset of parameters, or if we assume that some parameter values occur with a higher probability. Nevertheless, they give a first global picture of the potential effect of biocontrol. It is then important to identify the ranges of parameters for which biocontrol has an important effect, i.e. the agroecological contexts in which biocontrol would be significant.

**Figure 3.**
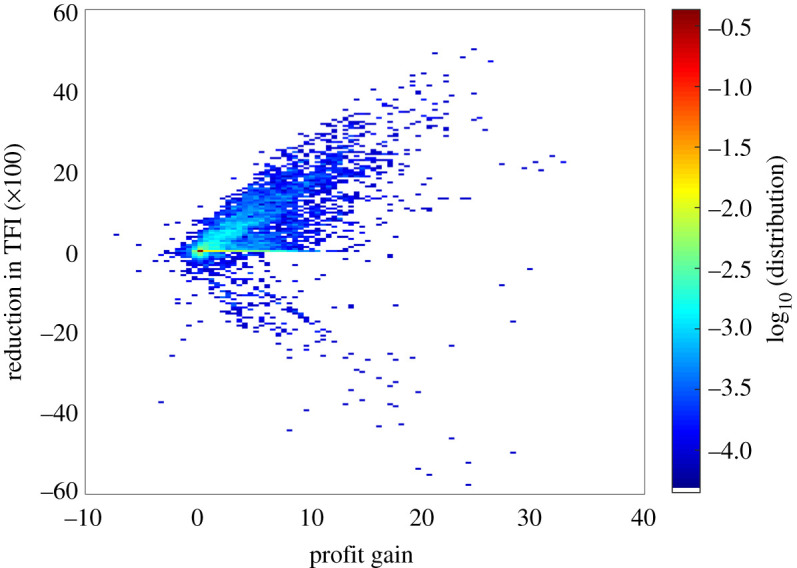
Bivariate distribution profit gain vs reduction in TFI, compared with an absence of biocontrol.

*Trade-off analysis.* We present in figure 3 the bivariate distribution of the profit gain and reduction in TFI, again computed over the whole set of parameters.

First, in 6% of the simulations, biocontrol has a significant effect on profit gain (in the following sense: |Δπ| is larger than 5% of the mean profit) and in 25% of the simulations, it has a significant effect on TFI reduction (|ΔTFI| is larger than 5% of the mean TFI). Altogether it has a significant effect on at least one of the two dimensions in 26% of the simulations. On the other hand, in 74% of the simulations, biocontrol has no significant effect, neither on profit nor pesticide use reduction.

For simulations in which the effect is significant, we observe two main trends, with a part of the distribution (upper diagonal) corresponding to a simultaneous positive effect of biocontrol on the profit gain and on the reduction in TFI. In this part of the distribution, we note a strong positive correlation between Δπ and ΔTFI (Pearson linear correlation coefficient 0.83). Another part of the distribution (lower diagonal, Pearson correlation coefficient −0.45) corresponds to a positive effect of biocontrol on profit gain, but a negative effect on the reduction of TFI (pesticide use is increased with respect to the scenario without biocontrol in this case). Still, among the simulations where we observed the significant effect, two-thirds of the distribution (66%) lies in the positive quadrant with a positive effect of biocontrol on both dimensions.

We looked for typical parameters that lead to

(i) a significant win–win biocontrol, with both large private gains (profits) and large public gains (pesticide use reduction). In that respect, we computed the modal parameter values (most frequent parameter value) corresponding to the top-right region in figure 3, corresponding to the 10% highest profit gain (Δπ>15) and 10% highest reduction in TFI (100×ΔTFI>15). In this window, we obtained the following modal values, for respectively $\left(\bar{q},s_{q},f,D_{P},D_{N},r_{P},\alpha ,\rho \right)$: $\left(0.3Q_{\max},0.3Q_{\max},1,1/n^{2},0.1/n^{2},\ln(4),4/3,\ln(4)\right)$. This corresponds to areas of relatively low agricultural potential, a highly fragmented and variable landscape, and a poorly efficient pesticide. From an ecological point of view, biocontrol seems to be favoured by a pest that grows relatively slowly and is highly mobile, but a predator relatively less mobile with a high predation rate.
(ii) a negative or not significant effect of biocontrol. The modal parameter values that lead to these situations (lower 10%): (Δπ<0 and 100×ΔTFI<−3) are $(0.3Q_{\max},0.1Q_{\max},-1,0.1/n^{2},0.1/n^{2}$, $\ln(10^{2}),5/6,\ln(4))$. This corresponds to a context with species that do not move a lot in a non-fragmented landscape.
(iii) a paradoxical effect of biocontrol, with private gains (increased profit) but public costs (increased pesticide use). Here, we looked for the modal parameter values that lead to a significant increase of profit gain (10% highest profit gain, i.e. Δπ>15) but a negative decrease in TFI (lower 10%, i.e. 100×ΔTFI<−3). We obtained the modal values: $(\bar{q},s_{q},f,D_{P},D_{N},r_{P},\alpha ,\rho )=(0.5Q_{\max},0.3Q_{\max},0,$ $1/n^{2},1/n^{2},\ln(10^{2}),4/3,\ln(4))$. This corresponds to highly variable agricultural landscapes, with mobile pest and predators, a high predation rate and a poorly efficient pesticide.

Some trends can be inferred from these parameter values: the win–win situations are characterized by different ecological parameters (higher pest diffusion, lower pest growth rate and higher effect of predation) and a higher fragmentation compared with the case where the effect of biocontrol is not significant. The lower diagonal (case 3) is rather characterized by the agronomic variables: a paradoxical effect of biocontrol tends to arise with a highly variable soil quality contexts. To get a better understanding of the interplay between these parameters, we conduct some statistical analysis.

*Effect of agronomic, ecological and economic parameters on the efficiency of biocontrol.* We denote by Θ the vector of parameters $\left(\bar{q},s_{q},f,D_{P},D_{N},r_{P},\alpha ,\rho \right)$. For each ecological, agronomic and economic parameter $\theta_{i}$, we define the marginal means associated with the value $\theta_{i}=y$

**Figure 4.**
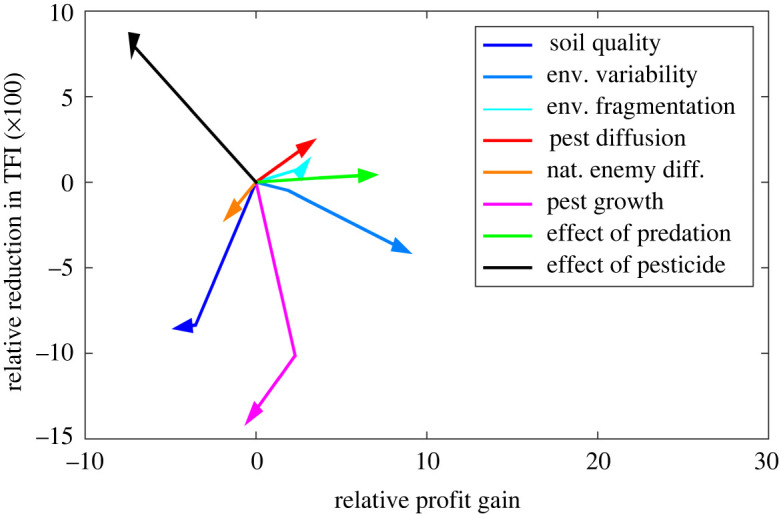
Marginal effects of the parameters. The arrows depict the variation in the biocontrol-induced profit gain and reduction in TFI, when a given parameter is increased, compared with when this parameter takes its lowest value. When an arrow points in the positive (respectively, negative) direction, it means that the effect of biocontrol is stronger (respectively, lower) when the parameter is increased.

3.1 $${\bar{\Delta \pi }}_{i}(y)={\text{mean}}_{\Theta \text{ s.t. }\theta_{i}=y}(\Delta \pi (\theta ))\;\text{and}\;{\bar{\Delta \mathrm{TFI}}}_{i}(y)={\text{mean}}_{\Theta \text{ s.t. }\theta_{i}=y}(\Delta \mathrm{TFI}(\theta )),$$

i.e. we compute the mean value of Δπ (respectively, ΔTFI) for the group corresponding to the focal parameter $\theta_{i}=y$, averaged across every level of the other variables. We plotted in figure 4 the variations of these quantities when y is increased: the lines connect the points $({\bar{\Delta \pi }}_{i}(y)-{\bar{\Delta \pi }}_{i}(y_{\min}),$ ${\bar{\Delta \mathrm{TFI}}}_{i}(y)-{\bar{\Delta \mathrm{TFI}}}_{i}(y_{\min}))$ when y varies within the range associated with the parameter $\theta_{i}$. The arrow points toward the direction of variation when y is increased. We thus get a simple summary of the marginal effect of each parameter on biocontrol. Note that a higher effect of biocontrol on a given outcome does not necessarily mean that this outcome will take on higher values when the parameter is increased. For instance, increasing a parameter may decrease the profit gain independently of biocontrol, but it can, in parallel, increase the effect of biocontrol.

Based on this figure 4, we deduce the following impact of the variables on the expected effect of biocontrol:

— Regarding the agronomic variables: increasing the soil quality reduces Δπ and ΔTFI; more variable quality maps lead to increased Δπ but lower ΔTFI; more fragmented quality maps lead to higher values of Δπ and ΔTFI.
— Regarding the ecological variables: larger pest diffusion leads to increased Δπ and ΔTFI; larger natural enemy diffusion leads to decreased Δπ and ΔTFI; larger pest growth leads to lower ΔTFI but the effect on Δπ is not clear. A larger interaction factor α (higher predation efficiency) leads to increased Δπ, but the effect on ΔTFI is less clear.
— Regarding the effect of the pesticide: increasing the pesticide-induced mortality rate leads to decreased Δπ and increased ΔTFI.

The effect of biological control is stronger in landscapes with a lower agronomic potential. The reason may be that in these landscapes NCH are more frequent as crop profit is lower. There are thus more natural enemies in the landscape when soil quality is low, everything else being equal. The interpretation of the effect of the soil quality variability (which was already observed in the trade-off analysis above) can be due to the fact that, in more heterogeneous landscapes, there are more plots of lower agronomic quality, which are likely to be used as NCH, favouring the natural enemy. In more fragmented landscapes, the pest tends to spend more times in NCH, which increases the effect of predation. This effect is even increased when the pest diffusion is high. Conversely, when the diffusion of the natural enemy is higher, though it may have easier access to the pest, it is also subject to detrimental effects due to the absence of resource during a part of the year and to the effect the pesticide.

When the pest growth rate is increased, even if the initial population is lower in the presence of the natural enemy, the farmer will often anticipate large pest pressure at the end of the period and use pesticide anyway, limiting the effect of biological control on the reduction of TFI. The effect of predation efficiency on biocontrol is less obvious than might have been expected. Although it leads to higher profits, thanks to reduced damage to crops, it has a limited effect on ΔTFI, meaning that it does not lead to a change in crop protection behaviour.

The effect of the parameter ρ (pesticide-induced mortality) on profit gain can be explained as follows. The more effective the pesticide is, the more farmers would favour its use, resulting in situations with low pest pressure and a high profit. The effect of biocontrol on profit is thus lower than in a situation with a less effective pesticide, in which it is used less and biocontrol has a more important role in pest regulation and crop protection. There is more room, however, to reduce pesticide use in a situation of a widely used efficient pesticide, predation being a substitute for the pesticide at the margin.

In general, when the effect of biocontrol on profit gain or reduction in TFI increases, this may either be due to a direct effect of the variable considered on the effectiveness of predation or to indirect effects caused by interactions with other variables and farmers' decisions.

*Linear regression model with interactions.* The trends in figure 4 correspond to the mean effect of each parameter, averaged over all other parameters. Due to interactions between parameters, these trends may not always reflect the real effect of the parameter, depending on the current context (i.e. the other parameter values). We checked the robustness of the above analysis with a linear regression model with two-way interactions (*stepwiselm* Matlab^®^ function, Statistics and Machine Learning toolbox; uses the *p*-value for an *F*-test of the change in the sum of squared error to add or remove terms in the model),

3.2 $$\Delta \pi =\beta_{0}+\sum_{i=1,\ldots ,8}\beta_{i}\theta_{i}+\sum_{i,j=1,\ldots ,8}\beta_{i,j}\theta_{i}\theta_{j}+\varepsilon ,$$

and similarly

3.3 $$100\Delta \mathrm{TFI}=\gamma_{0}+\sum_{i=1,\ldots ,8}\gamma_{i}\theta_{i}+\sum_{i,j=1,\ldots ,8}\gamma_{i,j}\theta_{i}\theta_{j}+\varepsilon .$$

Once fitted, the model (3.2) explains about one-half of the variations in Δπ, i.e. of the variations in the effect of biocontrol on profit ($R^{2}=0.48$). Regarding the TFI, the model (3.3) explains one third of the variations in ΔTFI ($R^{2}=0.34$). The parameter values $\beta_{i}$, $\beta_{ij}$, $\gamma_{i}$, $\gamma_{ij}$ are available in electronic supplementary material, S2, tables S2.1 and S2.2. The signs of $\beta_{i}$ and $\gamma_{i}$ inform us about the role of each parameter on the effect of biocontrol. These signs are mostly consistent with the findings in figure 4, with some exceptions. In particular, this approach shows that the effect of pesticide-induced mortality is complex and results from multiple interactions with other parameters. Its direct effect seems to increase the profit gain due to biocontrol, but it has negative interactions with all the other parameters (last line in electronic supplementary material, table S2.1, S2), which results in the overall negative effect observed in figure 4. It means that having an efficient pesticide tends to hamper the beneficial effect of the other parameters on profit, which may be caused by a lower pest pressure and a higher mortality of the natural enemy. Among these interactions, we observe a strong negative interaction coefficient between pesticide-induced mortality rate and predation efficiency (α): increasing α decreases the effect of ρ on profit gain and vice versa. Regarding ΔTFI, conversely, the interaction coefficients are mostly positive, resulting in an overall positive effect of pesticide-induced mortality on ΔTFI, although the intrinsic effect is negative ($\gamma_{i}<0$; see the last line in electronic supplementary material, table S2.2, S2). The antagonistic effect of soil quality variability that was observed above (positive effect on profit gain and negative effect on ΔTFI) is not observed through the linear coefficients $\beta_{i}$, $\gamma_{i}$, which are both positive. This global paradoxical trend seems to be caused by a highly negative interaction coefficient with the pest growth rate parameter: the decrease in predation effect observed when the pest has a high growth rate is reinforced in a landscape with strong variability, which leads to greater use of pesticides.

## 4. Discussion

In this paper, we developed a model that represents agricultural land-use and crop protection decisions, as well as the ecological dynamics of pests and their natural enemy. Our modelling approach provides a tool to assess biocontrol in a controlled framework, which offers several features.

First, by modelling the economic decisions of land use and pest control, we are able to assess effective biocontrol, i.e. the extra profit farmers derive from biocontrol and the social benefits corresponding to the reduction of pesticide use thanks to biocontrol. The ecosystem service of biocontrol is not only dependent on ecological conditions, but also on the economic behaviour of farmers who will turn a potential service into actual benefits, by reducing pesticide use. On the contrary, there may be no service of biocontrol in spite of an ecological function of pest predation if farmers use a lot of pesticide anyway, without accounting for the current pest pressure. In our model, profit-maximizing farmers endogenously account for the feedback effect of their decisions on the ecological dynamics. This leads to subtle results, such as the fact that a more effective pesticide leads to a lesser influence of biocontrol because farmers are using more of it, implying a smaller abundance of the natural enemy (which is harmed, too, by the pesticide).

As advocated in [16], obtaining such results is, of course, only possible with feedback models with spatio-temporal interactions between landscape, ecological processes and stakeholder decisions, as we developed in this study.

Second, there have been many attempts to quantify biocontrol from field data, but the results are often context-dependent and difficult to explain and generalize. A modelling approach can study biocontrol on a large range of contexts, providing insights on the agronomic and ecological conditions that are more likely to lead to efficient biocontrol. Our modelling exercise suggests that there are very few situations where biocontrol induces a profit loss and a majority of win–win situations (in two-thirds of our simulations with a significant effect), with often a positive correlation between profit gain and reduction in TFI. The use of biocontrol is, therefore, low risk for profit loss, and the situations that increase the efficiency of biocontrol over one dimension also tend to increase its effect on the other dimension. Nevertheless, we observed a very limited effect in many of the agroecological conditions we studied. For some conditions, however, biocontrol has a significant positive effect on private profit and/or reduction of pesticide use. Some rare situations can also lead to greater use of pesticides, particularly in environments with high soil quality and significant variability.

Some parameters seem to have a stronger effect on the magnitude of biocontrol. The effect of biocontrol seems to be more limited in areas with higher agricultural potential, or when the pest has a high growth rate. Other parameters seem to favour biocontrol. First, landscape fragmentation, which was already known in landscape ecology to increase the efficiency of predation [9,41], or a more mobile pest. In both cases, the pest will more likely encounter natural enemies in NCHs. Interestingly, there are many examples of species for which dispersal capacity is traded off against reproduction (‘D–R’ trade-off) [42,43]. Our study indicates that biocontrol is more efficient for pest species with a ‘D’ strategy. Scarab beetles are an example of pests with a relatively low reproduction rate, whose predators include ants, staphylinids and carabids [44].

These results offer prospects for empirical research. They emphasize the need for the joint analysis of parameters driving biocontrol in empirical studies. In particular, our simulations reveal some cross-effects that could be assessed in the field. For example, there seems to be a negative cross-effect between the efficiency of pesticide to regulate the pest and the efficiency of predation (increasing α decreases the effect of ρ on profit gain and vice versa). This effect comes in particular from the hypothesis of a non-specific pesticide, increasing the mortality rate of the two species in the same way. Although the mortality rate may, of course, be different for the two species, the predators involved in biocontrol are often very sensitive to pesticides [28]. More subtle interactions may also emerge, e.g. between soil quality variability and pest growth rate, with a negative cross-effect on pesticide reduction. Variability in itself (conversely to fragmentation, see figure 1 to observe the difference between these two descriptors of the soil quality map) is known to be beneficial to pest population growth in the absence of predation [45], which may explain this effect.

Of course, the determination of the ‘true’ effect of biocontrol should rely on an array of approaches, not just one. In this respect, bringing our approach in the game appears very useful. We point out that, beyond the results discussed above, we have developed a generic tool which can be exploited under other conditions which are not necessarily those of this study. The Matlab codes used to generate the analyses conducted here are available and fully commented at https://doi.org/10.17605/OSF.IO/Z2QCX.

These results, related to the effect of the agronomic context (land-use variability and fragmentation) on biocontrol, provide an indication on the changes that could be made in agrocosystem to increase biocontrol. In terms of future work, we see two interesting extensions for the economic part of the model. First, the model can be used to study how to influence land-use and pest control decisions through public policies. Incentives (pesticide taxation, NCH subsidies, or a price bonus for pesticide-free production) can be used to modify the economic context and lead to dynamic landscapes with a higher level of biocontrol and less pesticide use. Second, the model could be used to study the role of farmers’ agroecological knowledge on biocontrol. In the present version, farmers take their decisions according to the local pest density only. One could examine if accounting for the predator density (at local or at the landscape level), i.e. relying on agroecological knowledge, would improve the benefits from biocontrol.

## Data Availability

All of the Matlab codes that led to the results in this paper are available in the Open Science Framework repository: https://doi.org/10.17605/OSF.IO/Z2QCX. Supplementary material is available online [46].
